# Maternal deaths due to hypertensive disorders in pregnancy: Saving Mothers report 2002–2004

**Published:** 2007

**Authors:** J Moodley

**Affiliations:** Women’s Health and HIV Research Group, Nelson R Mandela School of Medicine, Durban

## Abstract

Hypertensive disorders of pregnancy (proteinuric hypertension, eclampsia, chronic hypertension, HELLP syndrome) are the commonest direct causes of maternal deaths in South Africa. Six hundred and twenty-eight (19.1%) of the 3 406 maternal deaths in a three-year period (2002−2004) were associated with hypertensive disorders of pregnancy. Cerebral complications, mainly cerebral haemorrhage, were the pathological causes of death in approximately 50% of the cases and eclampsia (convulsions in pregnancy associated with high blood pressure) was the commonest clinical condition leading to death from hypertension.

Avoidable factors, missed opportunities and substandard care in all three categories of patient-related, administrative, and healthcare-related components were found by the assessors of the National Committee on Confidential Enquiries into Maternal Deaths to be prevalent in the majority of the deaths. Prevention of complications by lowering high blood pressure fairly rapidly, early referral of high-risk cases to experts in the field, and the proper use of resuscitation skills should reduce maternal mortality associated with hypertensive disorders of pregnancy.

## Summary

Hypertensive disorders of pregnancy (HDP) are common conditions in South Africa. It has been reported that 18% of all admissions to King Edward VIII Hospital, Durban, consist of HDP^1^ and that 12% of all pregnancies in the Durban metropolitan area are associated with hypertension.^2^ It is therefore not surprising that complications associated with HDP are common, not only in the province of KwaZulu-Natal, but also in the rest of the country.^3^ The National Committee on Confidential Enquiries into Maternal Deaths (NCCEMD) is a committee that was established by the national Minister of Health to collate information obtained from maternal deaths throughout the country, and make recommendations in such a manner as to lead to improvements in maternal and neonatal care that would subsequently reduce the number of maternal deaths.

## Methods

The process involves notification of all maternal deaths by the head of a health institution once a discussion of the events surrounding the death has occurred. A structured maternal death notification form is completed and sent to the provincial maternal women and child health directorate, from whence it is sent to assessors. All the assessments are done by a pair of experienced clinicians, usually a doctor and a midwife. The assessment of each death is based on avoidable factors, missed opportunities and substandard care.

The data obtained should not be considered as those from a pure epidemiological study because information such as home deaths and deaths occurring in the wards of disciplines outside obstetrics and gynaecology is often missing, but it allows the assessment of patterns of deaths and provides trends from which recommendations could be suggested. The NCCEMD provides a collation of information arising from all maternal deaths over a three-year period called ‘Saving Mothers’.

## Report on hypertensive deaths

Hypertensive disorders in pregnancy are the commonest direct causes of maternal deaths in South Africa. In the latest Saving Mothers report 2002−2004, there were 628 deaths associated with HDP (chronic hypertension, proteinuric hypertension or pre-eclampsia, eclampsia), HELLP syndrome (haemolysis, elevated liver enzymes and low platelets) and rupture of the liver. Hypertensive disorders contributed to 19.1% (628 of 3 604) of maternal deaths in the triennium.^3^

The primary obstetric causes of deaths from hypertension in the last two reports are shown in Table 1. Eclampsia and proteinuric hypertension accounted for the majority of the deaths, namely 83% in the second triennium. Table 2 shows the final and contributory causes of maternal deaths. There was a decrease in deaths from pulmonary oedema and cardiac failure, compared to the 1999−2001 data. Deaths from renal failure also declined. It is surprising that deaths due to cerebral complications remained the same; they accounted for 50% of all hypertensive deaths.

**Table 1:** Primary Obstetric Causes Of Death In The Subcategories

*Subcategories*	*1999−2001*		*2002−2004*	
	*n*	*%*	*n*	*%*
Chronic hypertension	24	4.7	37	5.9
Proteinuric hypertension	139	27.4	171	27.2
Eclampsia	289	57	347	55.3
HELLP syndrome	44	8.7	70	11.1
Rupture of the liver	8	1.6	3	0.5
Acute fatty liver	3	0.6	0	0.0
Total	507		628	19.1

**Table 2:** Final And Contributory Causes Of Maternal Deaths For Hypertension And A Comparison With 1999−2001

*Organ system*	*1999−2001 deaths*		*2002−2004 % of deaths*	
	*n*	*%*	*n*	*%*
Hypovolaemic shock	39	7.7	49	7.8
Septic shock	18	3.6	16	2.5
Respiratory failure	81	16.0	155	24.7
Cardiac failure	179	35.3	89	14.2
Pulmonary oedema	17.2		
Cardiac arrest	18.9		
Renal failure	90	17.8	88	14.8
Liver failure	38	7.5	31	4.9
Cerebral complications	255	50.3	316	50.3
Metabolic complications	6	1.2	7	1.1
DIC	57	11.2	89	14.2
Multi-organ failure	65	12.8	104	16.6
Immune system failure	8	1.6	18	2.9
Unknown	2	0.4	56	8.9

All provinces reported deaths due to HDP and the Free State, Gauteng and Eastern Cape showed an increase in absolute numbers of deaths, but the proportions remained the same because of the actual increase in the total number of deaths. KwaZulu-Natal reported fewer deaths compared to the previous report (Table 3). Hypertensive disorders in pregnancy occurred in all age groups but 72 of the 105 deaths due to eclampsia (Table 4) occurred in the age group 20 years or younger, while 14 of the 32 women in the age group 40 to 44 years had eclampsia. Table 5 shows the age and parity in comparison with a general pregnant population.

**Table 3:** Deaths Due To Hypertensive Disease Per Province

*Province*	*1999−2001*		*2002−2004*	
	*Province*	*Number of deaths reported*	*% of deaths reported*	*Number of deaths reported*	*% of deaths reported*
Eastern Cape	75	28.6	89	24.1
Free State	58	23.3	100	23.1
Gauteng	85	20.0	137	20.5
KwaZulu/Natal	147	21.3	99	13.7
Limpopo	27	18.8	48	17.1
Mpumalanga	35	13.8	40	13.7
North West	38	18.5	53	16.3
Northern Cape	15	24.2	19	17.9
Western Cape	27	23.3	43	20.8
Total	507	21.1	628	18.4

**Table 4:** Age Distribution And Death Due To Hypertension In Pregnancy

*Category*	*< 20*	*20−24*	*25−29*	*30−34*	*35−39*	*40−44*	*45+*	*Unknown*	*Total*
Chronic hypertension	0	4	5	9	8	7	4	0	37
Proteinuric hypertension	28	39	42	32	18	9	3	0	171
Eclampsia	72	85	66	71	34	14	4	1	347
HELLP	5	15	21	23	4	2	0	0	70
Liver rupture	0	1	0	2	0	0	0	0	3
Acute fatty liver	0	0	0	0	0	0	0	0	0
Total	105	144	134	137	64	32	11	1	628

**Table 5:** Comparison Of Age And Parity With A General Pregnant Population

*Age*	*n*	*%*	*% gen pop*	*Parity*	*n*	*%*
< 20	105	16.7	10.5	0	287	45.7
20−24	144	22.9	26.5	1	116	18.5
25−29	134	21.3	24.1	2	85	13.5
30−34	137	21.8	20.2	3	52	8.3
35−39	64	10.2	12.0	4	30	4.8
40−44	32	5.1	5.2	5	13	2.1
45+	11	1.8	1.5	6+	15	2.4

Deaths from HDP occur at all levels of healthcare provision with a considerable proportion still occurring in level 1 hospitals. Table 6 shows avoidable factors, missed opportunities and substandard care. Patient-avoidable factors, namely, non-attendance for antenatal care (23% of cases with avoidable factors), infrequent attendance (6.9%), and delay in seeking help (26.7%) remain major problems and were identified in 524 of 628 deaths due to HDP.

**Table 6:** Avoidable Factors, Missed Opportunities And Substandard Care For Hypertension, And Comparison With 1999−2001

*Category*	*Avoidable factors in assessable cases*			
	*1999−2001*		*2002−2004*	
	*n*	*%*	*n*	*%*
Patient orientated	205	50.6	250	47.7
Administrative factors	329	74.3	225	39.3
Health-worker orientated				
Emergency management problems				
Level 1	116	68.2	218	65.3
Level 2	148	74.4	149	51.7
Level 3	91	49.5	77	35.6
Resuscitation problems	95	26.2	148	27.5

Table 7 shows an increase in avoidable factors in assessable deaths in respect of resuscitation in HDP (from 19.9% in 1999−2002 to 24% in 2002−2004). A high percentage of cases still do not have their circulatory systems corrected and airways secured. Overall, despite slight improvements, resuscitation techniques and methods are vital skills lacking during obstetric emergencies and should not only be emphasised in all clinical protocols of management, but also focused on during undergraduate and postgraduate training.

**Table 7:** Healthcare Provider Problems With Resuscitation In Hypertension And A Comparison With 1999−2001

*Description*	*1999−2001*				*2002−2004*			
	*Avoidable factor in assessable deaths*		*Distribution of avoidable factor per level of care*		*Avoidable factor in assessable deaths*		*Distribution of avoidable factor per level of care*	
	*n*	*%*	*n*	*%*	*n*	*%*	*n*	*%*
Resuscitation	101	19.9			151	24.0		
Airway not secured			23	22.8			34	22.5
Circulation not corrected			40	39.6			55	36.4
Inappropriate drugs given			6	5.9			3	2.0
Incompletely investigated			18	17.8			18	11.9
Not appropriately monitored			14	13.9			7	4.6

## Discussion

Hypertensive disorders of pregnancy and their complications remain the commonest direct causes of maternal deaths in South Africa. Furthermore, eclampsia constitutes the commonest primary cause of hypertensive-related deaths. It is of extreme concern that despite widespread provision of clear clinical protocols of management of severe pre-eclampsia/eclampsia countrywide, intracerebral haemorrhage remains the commonest final cause of death due to HDP. This once again implies that due attention is not being placed on the lowering of very high blood pressure levels, or that there is a lack of continued monitoring of blood pressure values during the referral period, labour and postpartum period. Health professionals must learn to lower acute severe blood pressure levels in young pregnant women on admission.

Severe pre-eclampsia is associated with blood pressure values which fluctuate; there are peaks and troughs during the acute phase and unless the high blood pressure is monitored frequently and lowered fairly rapidly but smoothly, complications are likely to occur in both mother and baby, namely, cerebrovascular accidents, convulsions and abruptio placenta. The characteristics of high blood pressure in acute pre-eclampsia are different from the non-pregnant state. As mentioned, there are fluctuations and a change in diurnal variation, therefore blood pressure control is essential in young pregnant women. This is a clinical problem that occurs in other countries as well. In the current report from the UK, Why Mothers Die, cerebral haemorrhage was also the commonest cause of death in the HDP and a similar recommendation was made with regard to the need to lower very high systolic blood pressures.^4^

A problem that is highlighted in the latest Saving Mothers report is the increasing number of adverse events in the postpartum period.^3^ In the last report (1999−2001) it was documented that a constant avoidable factor was the lack of monitoring in the antenatal period during labour and particularly the postpartum period.^5^ It must be emphasised that monitoring of vital signs must be performed frequently at all times in the acute phase of the condition. In practical terms, this implies that patients need to have their blood pressure, pulse rate, respiratory rate, Glasgow coma scale (GCS), fluid balance, urinary output and blood coagulation parameters measured regularly.

Automatic blood pressure machines, which are used widely (even in South Africa) need to be checked regularly as they tend to underestimate blood pressure values.^6^ Antihypertensive therapy must be instituted early and not stopped abruptly, but rather the dosage decreased in a step-down fashion.^1^ Most importantly, health professionals must be made aware of the fact that delivery of the woman with severe pre-eclampsia/eclampsia does not mean cure of the disease and that complications are unlikely to occur in the immediate postpartum period. In fact, the latest Saving Mothers report shows an increasing number of deaths associated with eclampsia in the postpartum period.^3^ Women with severe HDP *must* be managed in a high-dependency area or, if this is unavailable, an area set aside in any general ward can be used for this purpose and monitoring done at least every 30 minutes for the first 24 hours post delivery.

Teenage pregnancy remains a major problem. Eclampsia seems to have a predilection for this age group, or it may be an age group who delay enrolling for antenatal care, or have socalled ‘hidden’ pregnancies. A significant proportion of women younger than 24 years contributed to deaths from eclampsia, and a significant proportion again had no antenatal care, or infrequent attendance. The previous Saving Mothers reports recommended that contraceptive services and information on termination of pregnancy need to be made freely available and accessible. This is obviously not occurring. This recommendation is made again, and in addition, the involvement of communities, schools, technikons and universities in spreading the information about this problem through newsletters, lectures and open forums must be considered. Deans, university principals and heads of midwifery colleges should become involved in disseminating information.

Two factors in the current Saving Mothers report that need further investigation, monitoring and comment are the decline in: (1) deaths from HDP in KwaZulu-Natal; and (2) deaths from pulmonary oedema. There may be contradictory messages in these findings. Firstly, protocols for appropriate fluid balance might be working; this is also indicated by the decline in deaths associated with renal failure. It probably indicates better fluid balance management. On the other hand, there were more deaths from respiratory and multi-organ system failures. The decline in deaths from HDP in KwaZulu-Natal is difficult to explain and requires an in-depth review of the management of hypertensive disorders in this province. Conversely, there appears to be an increase in hypertensive deaths in the Free State and Gauteng. This may be due to better reporting, but these provinces have always provided quality maternal death notification reports.

The UK has seen a decline in deaths from HDP from 264 in their triennial reports in the 1950s down to 14 deaths in the sixth Why Mothers Die report.^4^ This was probably achieved by: (1) promoting antenatal care and instituting a recall system for defaulters; (2) instituting regional centres and regional obstetricians to provide advice on, or caring for women with severe pre-eclampsia/eclampsia; and (3) educating health professionals through audits and involving the general public about the dangers of pre-eclampsia.

Antenatal attendance and transport delays remain challenges, and community education on a continuing basis must be made a priority. Antenatal care free of financial charge does not appear to solve the problem of attendance. It is known that women confirm their pregnancies at an early stage in pregnancy by attending general practitioner rooms or clinics.^7^ A breakdown in continued care is apparent, patients do not seek antenatal care or general practitioners do not provide advice and continuing antenatal care. Due attention should be given to maternity care in continuing professional education for general practitioners, and shared care between general practitioners and health providers should be considered. Further, more emphasis on antenatal care and contraceptive services must be emphasised in healthcare educational curricula. This information should be brought to the attention of all heads of educational institutions.

The finding of high levels of avoidable health worker-orientated problems, particularly at level 1 hospitals, is extremely disturbing. It may imply that teaching at undergraduate level and during internship is of a poor quality and/or does not concentrate on practical skills. Emergency resuscitation, failure to refer, and substandard care may indicate lack of protocols, but may also be due to the fact that community service doctors, interns, medical officers, etc, do not have the requisite skills. Therefore, more effort needs to be based on face-to-face, on-site education for this category of health worker. Further, the inclusion of special focused teaching on resuscitative skills in the undergraduate medical programme must be considered and brought to the attention of the Committee of Deans and similar bodies involved in healthcare professional training. In respect of patient-avoidable factors, contact must be made at the community level to heighten awareness of the advantages of antenatal care, through meetings in community halls, the radio and newspapers.

## Conclusion

In general, it is disappointing that many of the recommendations made in previous reports have not resulted in significant changes in avoidable factors in relation to patients, healthcare providers and administration. A greater commitment to the reduction of maternal deaths must be made by civil society (government, healthcare providers and the public at large) if pregnancy is to be made safer.

## Recommendations

Clinical guidelines must emphasise the need for:

● Proper control of very high blood pressure. Severe hypertension must be treated effectively and high blood pressure values controlled throughout treatment in hospital. The advice of an obstetrician experienced in the management of severe pre-eclampsia/eclampsia should be obtained as early as possible in the clinical management of these high-risk cases.

● Monitoring of vital signs must be done regularly in the antenatal, intrapartum and post-delivery periods. Monitoring must continue for at least 24 hours in a high-dependency area for all severe pre-eclamptics and eclamptics.

In addition:

● Maintain and strengthen referral patterns. Hand in hand with referral patterns is the need to improve transport services.

● Teenage pregnancies are at high risk. Contraceptive services need to be strengthened for teenagers and scholars. Information on the availability of termination of pregnancy services must be widely disseminated.

● Creation of the post of a regional reproductive health advisor/expert to promote and update protocols and to carry out staff training at all levels of healthcare is important.

● Improving public knowledge of the importance of antenatal care is essential.

● Motivating changes in medical and nursing curricula is imperative so that more emphasis is placed on practical training in emergency resuscitation skills, antenatal care and contraceptive education. This can be done through the HPCSA, the Committee of Deans and similar formal structures.

## Practice points

The dangers of very high blood pressure leading to cerebral haemorrhage need to be recognised by all health professionals and antihypertensive therapy prescribed timeously.

● Teenage pregnancies are at risk of eclampsia.

● Early-onset pre-eclampsia poses threats to the mother and foetus.

● MgS0_4_ is the anticonvulsant of choice – it is not an antihypertensive.

● There are a number of instances in which primary healthcare facilities failed to test for proteinuria and the women went on to develop pre-eclampsia. Basic tests (urine testing, BP measurement, weight gain and eliciting oedema) must be done at primary healthcare clinics. Patients and their families need to be educated about symptoms of pre-eclampsia.
